# What is the clinical evidence on psilocybin for the treatment of psychiatric disorders? A systematic review

**DOI:** 10.1097/j.pbj.0000000000000128

**Published:** 2021-02-11

**Authors:** Henrique Castro Santos, João Gama Marques

**Affiliations:** a Hospital Júlio de Matos, Centro Hospitalar Psiquiátrico de Lisboa; b Clínica Universitária de Psiquiatria e Psicologia Médica, Faculdade de Medicina, Universidade de Lisboa, Lisbon, Portugal.

**Keywords:** anxiety, depression, obsessive-compulsive disorder, psilocybin, substance-related disorders

## Abstract

**Background:**

Psilocybin is a predominant agonist of 5HT_1A_ and 5HT_2A/C_ receptors and was first isolated in 1958, shortly before it became a controlled substance. Research on the potential therapeutic effects of this compound has recently re-emerged alongside what is being addressed as a psychedelic renaissance.

**Methods:**

In this paper we performed a systematic review of the clinical trials conducted so far regarding the therapeutic effects of psilocybin on psychiatric disorders. The eligibility criteria included clinical trials that assessed psilocybin's potential therapeutic effects on patients with psychiatric disorders. Nine hundred seven articles were found and screened in regard to the title, from which 94 were screened through abstract and 9 met the eligibility criteria and were included.

**Results:**

The papers published focused on 3 disorders: depression, obsessive-compulsive disorder (OCD) and substance use disorder (namely tobacco and alcohol). Psilocybin has shown a relatively safe profile and very promising results, with reductions found on most of the psychiatric rating scales’ scores. Research on depression showed the most solid evidence, supported by 3 randomized controlled trials. Studies on OCD and substance use disorder showed more limitations due to their open-label design.

**Conclusions:**

Altogether, the results from the studies reviewed in this paper suggest a substantial therapeutic potential. This calls for further research to confirm the results observed so far and further explain the underlying mechanisms.

## Introduction

Psilocybin is a substituted indolealkylamine from the tryptamine group of compounds. Its main active metabolite, psilocin, is obtained after desphosphorylation of psilocybin in the intestinal mucosa.^1,2^ Psilocybin administration can lead to changes in perception, derealization, depersonalization, impaired attention, thought content disorder, symptoms of anxiety or elation and change of intuition.^3^ Psilocybin as well as psilocin are substances with predominant agonist activity on serotonin's 5HT_1A_ and 5HT_2A/C_ receptors,^2^ the latter being considered necessary for the hallucinogenic effects.^4^

Psilocybin can be found in over 100 species of mushrooms, many of them belonging to the genus Psilocybe,^5^ and their use dates back more than 3500 years in Mexico.^6^ However, psilocybin was first isolated only in 1958 by Albert Hofmann.^7^ Shortly after, it became marketed as *Indocybin;* however, clinical research on psilocybin was scarce, limited to anecdotal case-reports.^8,9^ As a result of the increasing widespread use of psychedelics in the 1960s, psilocybin research stopped almost completely after the Controlled Substances Act classified it as a Schedule I substance.^10^ Nevertheless, psychedelics, such as psilocybin, are one of the safest known classes of central nervous system drugs, showing a very low potential for addiction.^2,11,12^ A pooled analysis^13^ of 8 double-blind placebo-controlled studies with 110 healthy subjects receiving psilocybin suggested that the administration of moderate doses of psilocybin to healthy, high-functioning and well-prepared subjects in the context of a carefully monitored environment was associated with an acceptable level of risk. Given the risks associated with its use, Johnson and his colleagues have developed guidelines for research safety that include measures such as careful volunteer preparation and having a safe physical session environment.^12^

After decades of suspension, human hallucinogen research has resumed in what is being addressed as a Psychedelic Renaissance.^14^ In fact, by 2005, around 2000 subjects had undergone psychotherapy in clinical studies with psilocybin.^15^ In this paper, we review all the clinical trials conducted so far on the potential therapeutic effects of psilocybin on patients with psychiatric disorders.

## Methods

**Figure 1:**
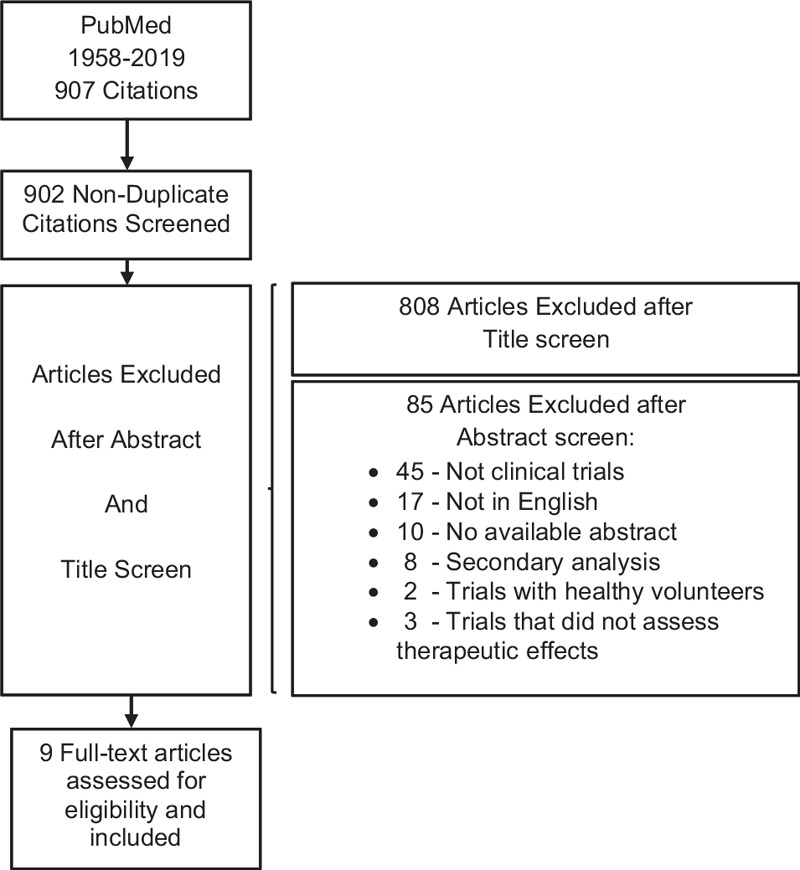
Flow of citations according to PRISMA (Preferred Reporting Items for Systematic Reviews and Meta).

The search was last performed in the PubMed database on the 5th of April 2019 for the term “psilocybin” on PubMed (search details: “psilocybin”[MeSH Terms] OR “psilocybin”[All Fields]). The eligibility criteria included clinical trials that assessed psilocybin's potential therapeutic effects on patients with psychiatric disorders. Studies were excluded if they were not in English, abstract was not available, they were studies with only healthy volunteers, they did not assess therapeutic effects, or they were secondary analyses. No further restrictions were imposed in regard to the type of intervention, outcomes, population of study or comparators. Nine hundred seven articles were found and screened in regard to the title, from which 94 were screened through abstract and 9 met the eligibility criteria and were included (see Fig. 1). A summary of the studies included is presented in Table 1.

**Table 1:** Summary of the studies included in this systematic review

Publication	Study design	Disorder/diagnosis	Sample	Intervention	Primary efficacy measures	Main findings
Grob et al 2011	Randomized controlled double-blind with crossover	Advanced-stage cancer and anxiety	n = 12	2 sessions several weeks apart:	BDI for depression	BDI score reduction at 6 month follow-up
				psilocybin (0.2 mg/kg)	Profile of Mood States Brief	STAI-T score reduction at 1 and 3 months follow up
				niacin (250 mg)	STAI-T and STAI-S for anxiety	
					BPRS	
					5-DASC	
Ross et al 2016	Randomized controlled double-blind with crossover	Depression and anxiety in life-threatening cancer	n = 29	2 dose sessions combined with psychotherapy, 7 weeks apart:	STAI-T and STAI-S for anxiety	Reductions on STAI-T, STAI-S, HADS-A, HADS-D, HAD-T and BDI 1 day, 2, 6 and 7 weeks post 1st psilocybin dose session
				psilocybin (0.3 mg/kg)	HADS-A for anxiety; HADS-D for depression; HAD-T	Within group reductions on anxiety and depression at every time point until the last one—26 weeks post dose 2
				niacin (250 mg)	BDI for depression	BDI 83% anti-depressant response vs 14% in the control group at 7 weeks post dose 1
					Remission and Response rates	HAD-A 58% anxiolytic response vs 14% in the control group at 7 weeks post dose 1
						BDI ∼85% of anti-depressant remission vs ∼15% in the control group at 7 weeks post dose 1
Griffiths et al 2016	Randomized controlled double-blind with crossover	Depression and anxiety in life-threatening cancer	n = 51	2 dose sessions + psychological support, ∼5 weeks apart:	GRID-HAMD-17 for depression	GRID-HAMD-17 higher response in high dose first group—92% vs 32% at the 5-week time-point after session 1
				psilocybin (1 or 3 mg/70kg)	HAM-A for anxiety	GRID-HAMD-17 higher remission in high dose first group—60% vs 16% at the 5-week time-point after session 1
				psilocybin (22 or 30mg/70kg)	Remission and Response rates	HAM-A higher response in high dose first group—76% vs 24% at the 5-week time-point after session 1
						HAM-A higher remission in high dose first group—52% vs 12% at the 5-week time-point after session 1
Carhart Harris et al 2016	Open-label, no control group	Treatment-resistant depression	n = 12	2 sessions combined with psychological support, 1 week apart:	QIDS for depression	QIDS score reduction at 1, 2, 3 and 5 weeks and 3 months post high dose session
				1st: psilocybin (10mg)	BDI for depression	BDI score reduction at 1 week and 3 months post high dose session
				2nd: psilocybin (25mg)	STAI-T for anxiety	STAI-T score reduction at 1 week and 3 months post high dose session
					SHAPS for anhedonia	SHAPS score reduction at 1 week and 3 months post high dose session
					HAM-D for depression	HAM-D mean score (SD) reduction at 1 week post high dose session
					MADRS for depression	MADRS mean score (SD) reduction at 1 week post high dose session
					GAF—Global Assession of Function	Increase on MADRS mean score (SD) at 1 week post high dose session
					Remission and Response Rates	8 of the 12 (67%) patients achieved complete remission at 1 week and 7 (57%) patients continued to meet criteria for response at 3 months
						GAF score increase at 1 week post high dose session
Carhart Harris et al 2017	Follow-up, Open-label, no control group	Treatment-resistant depression	n = 20	2 sessions combined with psychological support, 1 week apart:	QIDS-SR16	QIDS-SR16 score reduction at 1, 2, 3 and 5 weeks, 3 and 6 months post high dose session
				1st: psilocybin (10 mg)	BDI for depression	BDI score reduction at 1 week, 3 and 6 months post high dose session
				2nd: psilocybin (25 mg)	STAI-T for anxiety	STAI-T score reduction at 1 week, 3 months and 6 months post high dose session
					SHAPS for anhedonia	SHAPS score reduction at 1 week and 3 months post high dose session
					HAM-D for depression	HAM-D mean score (SD) reduction at 1week post high dose session
					GAF—Global Assession of Function	GAF score increase at 1 week post high dose session
Moreno et al 2006	Open-label, dose-escalation	Obsessive-compulsive disorder	n = 9	4 escalating dose sessions ≥ 1 week apart:	Yale-Brown Obsessive Compulsive Scale	Combined baseline mean YBOCS scores, when stratified by dose groups, reduced 24 hours after psilocybin administration
				1st psilocybin 100 μg/kg	Visual Analog Scale (VAS)	from a range of 18.3 to 24.1 to a range of 10.7 to 11.3
				2nd psilocybin 200 μg/kg		
				3rd psilocybin 300 μg/kg		
				psilocybin 25 μg/kg given randomly in a session after the 1st one		
Bogenshutz et al 2015	Open-label, no control group	Alcohol addiction	n = 10	2 sessions: at week 4 and at week 8, combined with psychotherapy	Timeline follow-back (TLFB) change in:	mean %HDD reduction on weeks 5–36 vs baseline
				psilocybin (0.3 mg/kg)	- Percent of Heavy Drinking Days (%HDD)	mean %HDD reduction on weeks 5–36 vs weeks 1–4
				psilocybin (0.4 mg/kg)	- Percent of Drinking Days (%DD)	mean %DD reduction on weeks 5–36 vs baseline
						mean %DD reduction on weeks 5–36 vs weeks 1–4
						Higher scores on HRS intensity subscale, 5D-ASC, and MEQ correlated with reduction on heavy drinking days
Johnson et al 2014	Open-label, no control group	Tobacco addiction	n = 15	3 sessions at 5, 7 and 13-week time points, combined w/psychotherapy:	Timeline follow-back (TLFB)	12 (80%) patients showed 7-day point prevalence abstinence at the 6-month follow-up
				1st: psilocybin (20 mg/70 kg)	Exhaled carbon monoxide (CO)	Reduction in mean self-reported daily smoking: ∼15 at intake vs ∼3 cigarettes a day at the 6-month follow-up
				2nd: psilocybin (30 mg/70kg)	Urinary cotinine	
				3rd: psilocybin (30 mg/70 kg)		
Johnson et al 2016	Follow-up, open-label, no control group	Tobacco addiction	n = 15	No intervention	Timeline follow-back (TLFB)	10 (67%) patients biologically verified as smoking abstinent at the 12-month follow-up
					Exhaled carbon monoxide (CO)	9 (60%) patients biologically verified as smoking abstinent at the long-term follow up (∼30 months post-TQD)
					Urinary cotinine	Reduction in TLFB at 10 weeks, 6 months, 12 months, and a mean of 30 months (long-term follow-up) post-TQD

## Results

### Depression

#### Anxiety and depression on life-threatening illness

On a randomized double-blind placebo-controlled crossover study,^16^ 12 patients with advanced-stage cancer and reactive anxiety were recruited. They underwent 2 sessions in a comfortable environment, spaced several weeks apart, during which they randomly ingested 0.2 mg/kg of psilocybin, on 1 session, and 250 mg of niacin on the other. The main goals were to establish cardiac safety [through blood pressure (BP) and heart rate (HR) control], evaluate subjective experience during the sessions (with the 5-Dimensional Altered states of Consciousness Scale^17^) and follow-up with the Beck Depression Inventory (BDI),^18^ the Profile of Mood States (POMS)^19^ and the State-Trait Anxiety Inventory (STAI)^20^ efficacy measures.

All 12 participants completed the 3 month-follow up, 11 completed the 4-month follow-up, and 8 completed the 6-month follow-up. The mean score for BDI decreased at the 6-month follow-up (BDI mean ∼7.4) in comparison to baseline (BDI mean = 16.1). The mean STAI Trait anxiety score was lower than baseline (mean STAI ∼43.0) after 1 month (mean STAI ∼34.0) and after 3 months (mean STAI ∼32.6). No significant changes were found in POMS mean scores. Compared to niacin, psilocybin increased the HR and BP (Table 2). Additionally, no adverse psychological effects were observed and all subjects tolerated well the treatment sessions.^16^

**Table 2:** Incidence of adverse effects attributable to psilocybin administration across the studies

Publication	Sample	Intervention	Blood pressure (BP) increase	Heart rate increase	Headaches	Nausea or vomiting	Transient anxiety	Transient psychotic-like symptoms	Other
Grob et al 2011	n = 12	2 sessions several weeks apart:	Mean peak systolic BP (SEM): 138.9 (6.4) mmHg	Mean (SEM) peak: 81.5 (5.8) bpm	–	–	–	–	No psychological adverse events registered
		psilocybin (0.2 mg/kg)	Mean peak diastolic BP (SEM): 75.9 (3.4) mmHg						
		niacin (250 mg)							
Ross et al 2016	n = 29	2 dose sessions combined with psychotherapy, 7 weeks apart:	“Non-clinically significant elevation” in 76%	28%	14%	17%	Transient thought disorder in 7%	–	
		psilocybin (0.3 mg/kg)	peak mean systolic BP 142 mmHg	Mean peak: HR 71 bpm				Paranoid ideation in 3%	
		niacin (250 mg)	peak mean diastolic BP 83 mmHg						
Griffiths et al 2016	n = 51	2 dose sessions + psychological support, ∼5 weeks apart:	Systolic BP (>160 mmHg): 34% in the high-dose session; 17% in the low-dose session	–	6% in the high-dose session	15% in the high-dose session	26% in the high-dose session	2% in the high-dose session	Physical discomfort: 21% in the high-dose session vs 8% in the low-dose session
		psilocybin (1 or 3 mg/70 kg)	Diastolic BP (>100 mmHg): 13% in the high-dose session; 2% in the low-dose session	–	0% in the low-dose session	0% in the low-dose session	15% in the low-dose session	0% in the low-dose session	Psychological discomfort: 32% in the high-dose session vs 12% in the low-dose session
		psilocybin (22 or 30 mg/70 kg)							
Carhart Harris et al 2016	n = 12	2 sessions combined with psychological support, 1 week apart:	–	–	33%	33%	100%	Transient paranoia in 8%	–
		1st: psilocybin (10 mg)						Transient thought disorder in 75%	
		2nd: psilocybin (25 mg)							
Carhart Harris et al 2017	n = 20	2 sessions combined with psychological support, 1 week apart:	–	–	40%	25%	75%	Transient paranoia in 15%	1 patient became transiently uncommunicative
		1st: psilocybin (10 mg)						Visual hallucinations in 70%	
		2nd: psilocybin (25 mg)							
Moreno et al 2006	n = 9	4 escalating dose sessions ≥ 1 week apart:	One subject (11%) experienced transient hypertension with peak BP values of 142/105 mmHg	–	–	–	–	–	–
		1st psilocybin 100 μg/kg							
		2nd psilocybin 200 μg/kg							
		3rd psilocybin 300 μg/kg							
		psilocybin 25 μg/kg given randomly in a session after the 1st one							
Bogenshutz et al 2015	n = 10	2 sessions: at week 4 and at week 8, combined with psychotherapy	Peak systolic BP: ∼150 mmHg	No significant changes	50%	10%	–	–	1 patient reported insomnia on the night following a psilocybin session
		psilocybin (0.3 mg/kg)	Peak dyastolic BP: ∼90 mmHg						1 participant experienced diarrhea during 1 psilocybin session
		psilocybin (0.4 mg/kg)							
Johnson et al 2014	n = 15	3 sessions at 5, 7 and 13-week time points, combined w/psychotherapy:	Systolic BP mean peak (SD): 153 (11) mmHg	Mean peak (SD): 68 bpm	80%	–	–	Transient thought disorder in 40%	–
		1st: psilocybin (20 mg/70 kg)	Diastolic BP mean peak (SD): 87(11) mmHg						
		2nd: psilocybin (30 mg/70 kg)							
		3rd: psilocybin (30 mg/70 kg)							
Johnson et al 2016	n = 15	No intervention	–	–	–	–	–	–	One participant showed decrease in well-being or life-satisfaction at the 12-month follow-up
									reportedly due to re-experiencing traumatic childhood memories

Another randomized double-blind placebo-controlled crossover study^21^ assessed the efficacy of a single dose of 0.3 mg/kg psilocybin vs 250 mg of niacin, both in combination with psychotherapy, to treat anxiety and depression in 29 patients with life-threatening cancer. The crossover occurred 7 weeks after dose 1 and patients were then followed for another 6.5 months. Primary outcome measures were assessed with self-reports on: STAI^20^ of State (STAI-S) and of Trait (STAI-T) subscales; Hospital Anxiety and Depression Scale (HADS)^22^ subscales of anxiety (HADS-A), depression (HADS-D) and total (HAD-T); and BDI.^18^ No serious adverse events were reported.

For the pre-crossover period, the psilocybin group showed statistically significant reductions on all 6 primary outcome measures at every time point, with large effect sizes (*d* ≥ 0.80). Similarly, at every time point after the second session, on every primary outcome measure, the psilocybin-first group reported significant within-group reductions on anxiety and depression scores. Clinically significant response rates were defined as a 50% or greater reduction in a score relative to baseline. Accordingly, at the 7-week time point post-dose 1, 83% of the subjects in the psilocybin-first group vs 14% in the niacin-first group met criteria for anti-depressant response (with BDI). Regarding anxiolytic response, for the same time point, 58% vs 14% (with HAD-A) and ∼75% vs ∼25% (with HAD-T) met criteria, favouring the psilocybin-first group. In addition, about 85% of psilocybin-first group vs ∼15% (with BDI) of the control group met criteria for anti-depressant remission, defined as 50% or greater reduction plus HADS-D ≤7^23^ or BDI ≤12.^24,25^ The assessment of the Mystical Experience Questionnaire (MEQ 30)^26^ at the end of the first session showed a significant positive correlation with the scores, from baseline to 6 weeks after dose 1, on HADS-T (Spearman's *r* = 0.39), HADS-A (*r* = 0.36), HADS-D (*r* = 0.30), BDI (*r* = 0.49), STAI-S (*r* = 0.42), STAI-T (*r* = 0.39).

Thirdly, a randomized double-blind crossover study^27^ compared the effects of a low dose (1 or 3 mg/70 kg) vs a high dose (22 or 30 mg/70 kg) of psilocybin on 51 patients with life-threatening cancer and symptoms of depression or anxiety. The doses were administered in 2 sessions, 5 weeks apart. Preparatory meetings were conducted to establish rapport. The drug sessions took place in a living-room-like environment where psychological support was available. The primary outcome measures chosen were the GRID-Hamilton Depression Rating Scale (GRID-HAM-D-17)^28^ and the Structured Interview Guide for the Hamilton Anxiety Rating Scale (HAM-A).^29^ A clinically significant response was defined as ≥50% score decrease compared to baseline. Symptom remission was defined as ≥50% reduction from baseline scores plus a score of ≤7 on either GRID-HAMD or HAM-A.^30,31^ Fifteen secondary measures were also applied, including BDI,^18^ HADS^22^ and STAI.^20^

No serious adverse events were reported although some adverse events were registered. 5 weeks after session 1, 92% in the high-dose-first group vs 32% in the low-dose-first group showed clinically significant responses, according to GRID-HAMD-17 scores. For the same time point and outcome measure, 60% vs 16% of the participants showed symptom remission, favouring the high-dose-first group. According to the HAM-A scores, the high-dose-first group also presented larger response rates (76% vs 24%) and remission rates (52% vs 12%) comparing to the low-dose-first group. Furthermore, MEQ30^26^ scores were substantially correlated with score reductions in HADS Anxiety (−1.50), HADS Depression (−1.11), HADS Total (−2.62), and HAM-A (−3.93).

### Treatment-resistant depression

An open-label study has investigated the feasibility and efficacy of psilocybin administration alongside psychological support on treatment-resistant depression.^32^ The study included 12 subjects with major depression of a moderate to severe degree (17+ on the 21-item Hamilton Depression Rating scale [HAM-D]), and no improvement after treatment with 2 adequate courses of antidepressants from distinct pharmacological classes, lasting at least 6 weeks within the current depressive episode. Every subject took an initial low dose of 10 mg and a high dose of 25 mg of psilocybin, 7 days apart. Previously, a 4-hour preparatory session with psychiatrists was provided. Dosing sessions took place in a pre-decorated room and patients were suggested to relax and listen to music while under supervision by 2 psychiatrists. To evaluate feasibility and safety, BP, HR, observer ratings of psilocybin's acute effects as well as the revised 11-Dimension Altered States of Consciousness questionnaire (11D ASC)^33^ were assessed. The primary outcome chosen for efficacy was the mean change in the severity of depressive symptoms, assessed with QIDS, from baseline to 1 week after the high-dose session. Additionally, the HAM-D, Montgomery-Åsberg Depression Rating Scale (MADRS),^34^ Global Assessment of Functioning (GAF),^35^ BDI,^18^ State-Trait Anxiety Inventory^20^ STAI-T (trait version), 16-item Quick Inventory of Depressive Symptoms [QIDS]^36^ and Snaith-Hamilton Pleasure Scale (SHAPS)^37^ were assessed.

One patient reported deterioration during the 3-month follow-up. However, mean QIDS scores were significantly lower from baseline to 1, 2, 3, 5 weeks and 3 months (final follow-up) after treatment. Maximum reduction was seen at 2 weeks, with a mean difference of −12.9 points compared to baseline. All patients showed reduced depression severity 1 week [mean BDI (SD) = 8.7 (8.4)] and 3 months [mean BDI (SD) = 15.2(11.0)] after the high dose session, in comparison to baseline [mean BDI (SD) = 33.7 (7.1)]. Taking into account the criteria for remission (BDI score of ≤9), 8 patients achieved complete remission 1 week after treatment. Furthermore, 7 patients continued to meet criteria for response (defined as a 50% BDI score reduction vs baseline) 3 months after treatment, from which 5 were still in remission at this point. STAI-T anxiety scores were significantly reduced 1 week [STAI-T mean score (SD) = 40.6(14.2)] and 3 months [STAI-T mean score (SD) = 54.8(14.5)] post high-dose session vs baseline scores [STAI-T mean score (SD) = 70.1(5.8)]. Similarly, SHAPS scores were significantly lower 1 week [mean SHAPS (SD) = 1.4(2.7)] and 3 months [mean SHAPS (SD) = 2.8(3.7)] post-treatment vs baseline [mean SHAPS (SD) = 7.5(3.7)]. HAM-D mean scores (SD) decreased from baseline [21.4(4.5)] to the 1-week follow-up [7.4(6.9)]. Significant reductions on mean MADRS (SD) [31.0 (5.0) at baseline to 9.7(9.8) 1 week post-treatment] were also reported alongside increases on Global Assessment of Function [50.3 (9.2) at baseline to 77.7(13.0) 1 week post-treatment].

Later, a 6-month follow-up study^38^ was conducted with a sample increase to 20 patients. Treatment with psilocybin was well tolerated and no serious adverse events were registered. 19 subjects completed all assessments. QIDS-SR16 scores were significantly decreased at all post-treatment time points, with maximum effect size seen at 5 weeks (−9.2, Cohen's *d* = 2.3), compared to baseline. BDI scores were significantly lower at 1 week (mean reduction = −22.7), 3 months (mean reduction = −15.3) and 6 months post-treatment (mean reduction = −14.9); STAI-T anxiety scores were significantly reduced 1 week (mean reduction = −23.8), 3 months (mean reduction = −12.2) and 6 months after treatment (mean reduction = −14.8); SHAPS anhedonia scores decreased 1 week (mean reduction = −4.6) and 3 months after treatment (mean reduction = −3.3); HAM-D scores dropped week post-treatment (mean reduction = −14.8); and GAF scores increased 1 week post-treatment (mean increase = +25.3). A significant reduction on the suicidality scores of QIDS-SR16 was seen 1 week (mean reduction = −0.9), 2 weeks (mean reduction = −0.85), 3 weeks (mean reduction = −0.8) and 5 weeks post-treatment (mean reduction = −0.7). Similarly, reductions were observed on the suicide item of the HAM-D, 1-week post-treatment (mean reduction = −0.95), at which point 16 of the 19 patients were scoring 0 and none was showing increase from baseline nor reaching the maximum score on this measure. After assessing relapse at 6 months, out of the 9 subjects that met response criteria at 5 weeks post treatment, 3 had relapsed, according to criteria of QIDS score of 6 or more.

### Obsessive-compulsive disorder (OCD)

On a modified double-blind study^39^ that was set to evaluate the safety, tolerability and the potential therapeutic effects of psilocybin in OCD, 9 subjects who met criteria for current OCD were recruited. Inclusion criteria included at least 1 “treatment failure”, defined as a lack of significant improvement after an adequate treatment course with a serotonin reuptake inhibitor (SRI) for at least 12 weeks. Subjects were required to have tolerated well at least 1 prior exposure to indole-based psychedelics and took psilocybin in a dose escalation protocol in up to 4 sessions. The doses used were 25 (very low dose [VLD]), 100 (low dose [LD]), 200 (medium dose [MD]), and 300 (high dose [HD]) μg/kg of body weight. LD, MD, and HD were administered in that order, whereas the VLD was given randomly in a double-blind fashion at any session after the first dose (LD) session. During sessions, subjects wore eyeshades and listened to a standardized set of music in the presence of trained sitters. In order to evaluate obsessive-compulsive symptom severity, Yale-Brown Obsessive Compulsive Scale (YBOCS) and a visual analog scale (VAS) were used. In addition, the Hallucinogen Rating Scale (HRS)^40^ was assessed and vital signs were monitored.

Two subjects discontinued their participation after the first session (LD) due to discomfort with hospitalization. Regarding efficacy, a significant main effect of time on YBOCS scores was found, but no significant effect of dose or interaction of time and dose. Mean YBOCS scores immediately before (baseline) and 24 hours after psilocybin ingestion (T-24h) for each dose were as follows: 25 μg/kg (VLD), baseline = 18.29, T-24h = 11.14; 100 μg/kg (LD), baseline = 24.11, T-24h = 10.67; 200 μg/kg (MD), baseline = 19.57, T-24h = 11.00; 300 μg/kg (HD), baseline = 18.83, T-24h = 11.33. The combined baseline mean YBOCS scores, after stratification by dose groups, was significantly reduced 24 hours after administration, from a range of 18.3 to 24.1 to a range of 10.7 to 11.3. The comparison of baseline vs post-ingestion VAS scores for all doses combined was also statistically significant; however, no significant effect of time or dose was found on VAS scores for all doses combined. HRS total score and each of its subscales, with the exception of volition, showed a statistically significant linear main effect of dose.

## Substance use disorder

### Alcohol

An open-label study^41^ investigated the acute effects of psilocybin and its preliminary efficacy and safety in 10 subjects diagnosed with active alcohol dependence. Volunteers were required to have had at least 2 heavy drinking days (defined as ≥5 drinks/day for males and ≥4 drinks/day for females) in the past 30 days, to not being under treatment and to express concern about their drinking habit. Participants underwent 14 sessions of manualized intervention, including 2 psilocybin sessions. The other sessions consisted of 7 motivational enhancement therapy sessions, 3 preparation sessions and 2 debriefing sessions. Participants had to be abstinent and not in alcohol withdrawal. The drug sessions took place in a living-room-like environment, in the presence of 2 therapists. On week 4, participants took 0.3 mg/kg of psilocybin and, on week 8, 0.4 mg/kg. To measure the psilocybin's acute effects, the HRS Intensity subscale,^40^ the 5D-ASC^17^ (which includes MEQ^26^) and a Monitor Session Rating Form^42^ were assessed. Moreover, after each psilocybin session, the Addiction Research Center Inventory (ARCI), 49-item version,^43^ was evaluated. To evaluate substance use, the past 3-month version of Short Inventory of Problems (SIP)^44^ and Breath Alcohol Concentration (BAC) were assessed. The primary efficacy outcome measure was change in percent heavy drinking days (%HDD) at baseline and at weeks 5 to 12, assessed with the Time-Line Follow-Back procedure. Heavy drinking days were defined as days during which participants consumed ≥5 standard drinks, if the participant was male, or ≥4 standard drinks if the participant was female. A standard drink was defined as containing 14 g of alcohol. An analysis of the percent drinking days (%DD), defined as days during which participants consumed any amount of alcohol, was also performed. In addition, the following measures were assessed: The Stages of Change Readiness and Treatment Eagerness Scale (SOCRATES 8A),^45^ the Alcohol Abstinence Self-Efficacy Scale (AASE),^46^ the Penn Alcohol Craving Scale (PACS)^47^ and the Profile of Mood States (POMS).^19^ Safety was controlled through vital signs.

One subject left the study with no justification provided and its data was not used. No serious adverse events were reported. Mean percent heavy drinking days (%HDD) dropped during weeks 5 to 12 (∼9%HDD) compared to baseline (∼35%HDD), and compared to weeks 1 to 4 (∼27%HDD)—during which only psychosocial treatment was provided. These %HDD reductions persisted from 13 to 24 weeks and from 25 to 36 weeks, compared to baseline values. The mean %HDD decrease observed at weeks 1 to 4 was not statistically significant, in comparison to baseline. On the other hand, the mean %DD showed significant reductions in every time point when compared to baseline (∼42%DD). For this measure, the mean (SD) reduction on weeks 5 to 12 was 27.2 (23.7) vs baseline, and 21.9 (21.8) vs weeks 1 to 4. The reductions reported on both mean %HDD and mean %DD, on weeks 1 to 4, were not significant in comparison to baseline. However, compared to weeks 1 to 4, these outcomes showed statistically substantial decreases at all time points. The only exception was %HDD at weeks 9 to 12, at which the reduction did not reach significance. Furthermore, significant correlations were found between measures of acute effects (HRS Intensity subscale, MEQ total and ASC summary score) and favorable changes regarding drinking, craving and self-efficacy (%HDD, %DD, PACS scores and AASE). In addition, higher scores on the HRS intensity subscale (*r* = −0.76), 5D-ASC (*r* = −0.89), and MEQ(*r* = −0.85) were correlated with fewer heavy drinking days.

### Tobacco

An open-label pilot study researched the safety and preliminary efficacy of psilocybin on treating tobacco addiction.^48^ This study included 15 subjects that smoked a minimum of 10 cigarettes a day, that had multiple unsuccessful quit attempts and that still showed desire to stop smoking. In a 15-week treatment course, subjects attended weekly preparatory meetings in the first 4 weeks and took psilocybin on weeks 5, 7 and 13. Nevertheless, the last session was optional. A moderate dose (20 mg/70 kg) was administered in the first session and a high dose (30 mg/70 kg) was provided in the other 2 sessions; however, participants were allowed to repeat the moderate dose instead. A Target to Quit Date (TQD) was set for the same day as the first psilocybin session. Participants underwent cognitive behavior therapy as well as preparation for the psilocybin sessions. Treatment also included 2 components of an effective group based smoking cessation therapy.^49^ In addition, integration meetings and weekly support meetings were conducted after the TQD for 10 weeks. Short daily phone calls were made to participants for 2 weeks post-TQD to encourage abstinence. The primary efficacy outcome measures were the self-reported Timeline follow-back (TLFB),^50^ for retrospective number of cigarettes smoked per day, and the biological markers exhaled carbon monoxide (CO) and urinary cotinine levels. Safety was evaluated through BP and HR monitoring during psilocybin sessions. Additionally, post-session States of Consciousness Questionnaire^42,51^ ratings of acute adverse psychological effects, next-day headache ratings and Visual Effects Questionnaire data were assessed.

Three patients did not undergo the third session and 1 participant chose the moderate dose for the second psilocybin session. The remaining followed the default doses for each session. No clinically significant adverse events occurred. According to the TLFB and confirmed by CO and urinary cotinine measures, out of the 15 participants, 12 (80%) showed 7-day point prevalence abstinence at the 6-month follow-up. One of these 12 participants self-reported quitting on the TQD and was biologically verified as abstinent at all attended meetings, but was unable to attend the third psilocybin session or provide CO and urine samples for weeks 6 to 10 post-TQD. The other 11 participants self-reported quitting smoking on the TQD and showed biologically confirmed smoking abstinence up to 10 weeks post-TQD. Three of these 12 patients reported self-corrected lapses (defined as any discrete instances of smoking post-TQD) and 1 participant reported a relapse (defined as smoking on 7 or more consecutive days post-TQD^52^), after 13 weeks of continuous abstinence. Further analysis showed substantial reductions in mean self-reported daily smoking: ∼15 at intake vs ∼3 cigarettes a day at the 6-month follow-up. For the 3 participants that tested positive for smoking at the 6-month follow-up, an analysis on their TLFB assessments also revealed a significant reduction, from a reported mean of 20 cigarettes/day, at intake, vs 14 at the 6-month follow-up. Post-hoc testing for linear contrast showed significantly higher confidence to abstain from administration to the 6-month follow-up, and also reported significantly reduced craving and temptation to smoke across all time points.

Later, a long-term follow-up^53^ was conducted on the same 15 subjects. The primary outcome measures chosen were the CO, urinary cotinine, the TLFB^50^ and a Persisting effects questionnaire. Out of the 15 subjects, 3 did not complete the long-term (at a mean of 30months post-TQD) follow-up and were confirmed as daily smokers at the 12-month follow-up. For the latter time point, 10 (67%) participants were biologically verified as smoking abstinent from which 8 self-reported continuous abstinence since the TQD. At the long-term follow-up, 9 (60%) participants were biologically confirmed as smoking abstinent, from which 7 reported continuous abstinence since their TQD. Furthermore, statistically significant reductions in the self-reported TLFB were observed, in comparison to intake, at 10 weeks, 6 months, 12 months, and at a mean of 30months (long-term follow-up) post-TQD as follows: from a mean (SD) of 16.5 (4.3) cigarettes per day (CPD) at study intake, to 1.4 (3.8) CPD at 10 weeks; 2.7 (5.5) CPD at 6 months; 3.3 (6.5) CPD at 12 months; and 4.3 (6.6) CPD at the long-term follow-up.

## Discussion

All studies included in this review have suggested that psilocybin has a favorable safety profile, being well tolerated in general (see Table 2). The most common adverse events reported were transient hypertension, anxiety and nausea, and headaches that were limited to the experimental sessions in the majority of cases. These events go in accordance with previous reports.^13,42,54,55^ The occurrence of adverse events was more often reported with higher doses of psilocybin^27^; however, out of all studies, there were no reports of serious adverse events and all adverse events were readily managed by the staff without the need of pharmacological intervention. The safety of psilocybin use is conditioned mainly by the individual's expectations and the surrounding environment, which explains the wide amplitude of subjective effects^4^ and the concern about the conditions under which drug sessions were conducted in many of the studies cited.

Regarding results, on depression and anxiety symptomatology, 4 studies^16,21,27,32^ consistently showed immediate and enduring anti-depressant and anxiolytic effects. Notably, results have shown BDI score reductions lasting as long as 6 months^16^ and reaching as high as 83% anti-depressant response and 85% remission rates (defined by BDI scores), 7 weeks following a single dose of psilocybin.^21^ Significant reductions were also reported on other depression measures such as QIDS, GRID-HAMD-17 or MADRS. Furthermore, significant evidence for anxiety symptom reduction was also observed on scales such as STAI and HAM-A, with reports of 76% and 52% of response and remission rates, respectively^27^ (defined by HAM-A scores). Regarding OCD, 1 open-label study has found symptomatic relief and significant reductions on YBOCS scores.^39,56–58^ On tobacco addiction, 2 open-label studies regarding the same experiment have showed abstinence on 80% of the subjects after 3 months and on 67% after 9 months from the final dose session. In addition, 60% were biologically verified as abstinent roughly 27 months after the last dose session. Nevertheless, these 2 studies did not differentiate the effects of moderate and high doses. In regard to alcohol abuse, the open-label study reported significant reductions on the percent of heavy drinking days and percent of drinking days to up to 28 weeks. However, it was not able to differentiate the effects of the 2 doses used. Although the level and quality of evidence for psilocybin on substance use disorder are low, the preliminary results published so far are promising and have motivated researchers to conduct larger controlled trials: a 50-participant study on psilocybin-facilitated smoking cessation treatment (NCT01943994), a 180-participant study on psilocybin-assisted treatment of alcohol dependence (NCT02061293), the first study on psilocybin-facilitated treatment for cocaine use (NCT02037126) and multiple studies on depression (NCT03775200, NCT03429075, NCT03715127, NCT03181529, NCT03380442, NCT03554174, NCT03866174) and OCD (NCT03356483, NCT03300947). Overall the results obtained across studies are impressive, given that few administrations show long lasting effects, well beyond the time-course of the acute drug effects. Moreover, the studies on different disorders have coherently shown a significant therapeutic effect.

The mechanisms underlying the effects measured are, however, yet to be confirmed and several explanations have been proposed. Given that the agonism of psilocybin on the 5-HT_2A_ receptor is well-established, there is evidence supporting that it plays a role on the therapeutic effects reported, especially on depression. Some authors^59^ propose that one of the mechanisms through which psilocybin improves depression symptoms is by blocking the activity of inflammatory cytokines, namely TNF-α, whose levels have been found to be significantly higher in depressed patients.^60^ Research with fMRI in healthy volunteers after psilocybin administration has shown reduced activity in the medial Pre-Frontal Cortex (PFC) and decreased connectivity within the default mode network (DMN).^61,62^ This becomes more relevant given that depressive symptoms have been associated with increased activity in the medial PFC^63,64^ and that activity normalization of medial PFC has been shown with anti-depressant treatment.^65–67^ In the last paper regarding fMRI studies in patients with treatment-resistant depression under psilocybin treatment,^68^ increased DMN connectivity was observed 1 day after psilocybin administration. The authors proposed that, after psilocybin administration, DMN connectivity is reduced acutely and normalized afterwards with mood improvements, in a sort of “reset” mechanism. In the same study,^68^ a significant relationship was observed between reductions in amygdala cerebral blood flow (CBF) and reductions in depression symptomatology after psilocybin administration. Moreover, psycho-spiritual mechanisms have been proposed and explored before,^51,69^ as well as on several studies^21,27,41,53^ included in this review. Significant correlations have been found between mystical-type experiences and the outcomes. These mystical-type experiences are defined by feelings of positive mood, sacredness, a noetic quality, transcendence of time and space and ineffability.^42^ In one of the trials^27^ the correlations were still significant when the overall psilocybin effects’ intensity was controlled in a partial correlation analysis, suggesting that mystical-type experiences *per se* play an important role, independent from the overall intensity of psilocybin effects. A mediation analysis also suggested that mystical-type experiences mediated the therapeutic effects of psilocybin.

Regarding limitations, generalizability is limited since it includes 6 open-label studies. When it comes to OCD, the single open-label study's results^39^ should be analyzed carefully. Because even the lowest dose of psilocybin produced significant symptom reduction, this means that either there is a placebo effect present that cannot be measured for lack of a true placebo, or that psilocybin can be effective in such a low dose. Thus, further research on the effects of psilocybin in this disorder should try to clarify this by comparing it with a true placebo or a non-psychedelic active comparator. Furthermore, authors could not find a clear dose-response relationship to the change in YBOCS score nor correlation between YBOCS score reduction and the perceived psychedelic intensity. Additionally, the dose escalation protocol, which was also conducted on the tobacco addiction study, may have contributed to expectancy bias in both subjects and staff. Moreover, the need for staying at the hospital overnight may have introduced bias by selecting patients that could tolerate hospitalization. On alcohol dependence^41^ study, the lack of biological verification of alcohol use would decrease the bias risk of self-reported-only measures if conducted. Another limitation refers to the significant number of subjects across studies that reported previous psychedelic use, which contributes to expectancy bias. On the other hand, because psilocybin produces highly discriminable effects, blinding becomes a challenge, thus increasing the bias risk. The best option seems to be the use of an inactive low dose of psilocybin, as it showed some protection against monitor expectancy and it assures the benefit of the instruction that psilocybin is going to be administered on each session.^27^ Additionally, because psilocybin administration was associated with psychological support on most studies, it is not possible to make strong inferences regarding the extent of the effects. Nevertheless, the possibility of a synergistic interaction between the psilocybin administration and the psychological support is likely and should be explored.

Further trials with larger samples should be sought to confirm the results found so far, including research on the mechanisms underlying the effects reported. Altogether, the results from the studies reviewed in this paper suggest a very promising therapeutic potential from psilocybin. The results obtained so far, alongside the need for more effective psychiatric treatment, justify a call for further research.

## Author contributions

Both authors, Henrique Castro Santos and João Gama Marques, have contributed to the study conception and design, including material preparation, data collection and analysis. The first draft of the manuscript was written by the first author and both authors commented on every version of the manuscript. Both authors read and approved the final manuscript.

## Financial support and sponsorship

None.

## Presentation

None.

## Conflicts of interest

None.

## Abbreviations

Abbreviations: %DD = percent of drinking days, %HDD = percent of heavy drinking days, 11D ASC = 11-dimension altered states of consciousness questionnaire, 5-DASC = 5-dimension altered states of consciousness questionnaire, 5HT = 5-hydroxytryptophan, AASE = alcohol abstinence self-efficacy scale, ARCI = addiction research center inventory, BAC = breath alcohol concentration, BDI = beck depression inventory, BP = blood pressure, CBF = cerebral blood flow, CO = carbon monoxide, CPD = cigarettes per day, DMN = default mode network, fMRI = functional magnetic ressonance imaging, GAF = global assessment of function, GRID-HAM-D-17 = 17-item grid-hamilton depression rating scale, HADS = hospital anxiety and depression scale, HADS-A = hospital anxiety and depression scale subscale of anxiety, HADS-D = hospital anxiety and depression scale subscale of depression, HADS-T = hospital anxiety and depression scale total, HAM-A = hamilton anxiety rating scale, HAM-D = hamilton depression rating scale, HD = high dose, HR = heart rate, HRS = hallucinogen rating scale, LD = low dose, MADRS = montgomery-åsberg depression rating scale, MD = medium dose, MEQ 30 = 30-item mystical experience questionnaire, OCD = obsessive compulsive disorder, PACS = penn alcohol craving scale, PFC = pre-prontal cortex, POMS = profile of mood states, QIDS = quick inventory of depressive symptoms, QIDS-SR16 = 16-item quick inventory of depressive symptoms-self report, SD = standard deviation, SEM = standard error of the mean, SHAPS = snaith-hamilton pleasure scale, SIP = short inventory of problems, STAI = state-trait anxiety inventory, STAI-S = state-trait anxiety inventory of state, STAI-T = state-trait anxiety inventory of trait, TLFB = timeline follow-back, TNF-α = tumor necrosis factor alpha, TQD = target to quit day, VAS = visual analog scale, VLD = very low dose, YBOCS = yale-brown compulsive scale.
